# SARS-CoV-2 Omicron-neutralizing memory B-cells are elicited by two doses of BNT162b2 mRNA vaccine

**DOI:** 10.1126/sciimmunol.abn8590

**Published:** 2022-02-03

**Authors:** Ryutaro Kotaki, Yu Adachi, Saya Moriyama, Taishi Onodera, Shuetsu Fukushi, Takaki Nagakura, Keisuke Tonouchi, Kazutaka Terahara, Lin Sun, Tomohiro Takano, Ayae Nishiyama, Masaharu Shinkai, Kunihiro Oba, Fukumi Nakamura-Uchiyama, Hidefumi Shimizu, Tadaki Suzuki, Takayuki Matsumura, Masanori Isogawa, Yoshimasa Takahashi

**Affiliations:** ^1^ Research Center for Drug and Vaccine Development, National Institute of Infectious Diseases; Tokyo 162-8640, Japan.; ^2^ Department of Virology I, National Institute of Infectious Diseases; Tokyo 162-8640, Japan.; ^3^ Tokyo Shinagawa Hospital; Tokyo, 140-8522, Japan.; ^4^ Department of Pediatrics, Showa General Hospital; Tokyo, Japan; ^5^ Department of Infectious Diseases, Tokyo Metropolitan Bokutoh Hospital; Tokyo, Japan; ^6^ Department of Respiratory Medicine, JCHO Tokyo Shinjuku Medical Center; Tokyo, Japan; ^7^ Department of Pathology, National Institute of Infectious Diseases; Tokyo 162-8640, Japan.

## Abstract

Multiple SARS-CoV-2 variants possess mutations in the spike receptor-binding domain (RBD) with potential to evade neutralizing antibody. In particular, the Beta and Omicron variants escape from antibody neutralizing activity in those who received two doses of BNT162b2 mRNA vaccine. Nonetheless, boosting with a third vaccine dose or by breakthrough infection improves the overall breadth of the neutralizing antibodies, but the mechanism remains unclear. Here, we longitudinally profiled the cellular composition of RBD-binding memory B cell subsets and their antibody binding and neutralizing activity against SARS-CoV-2 variants following the second dose of mRNA vaccine. Two doses of the mRNA vaccine elicited plasma neutralizing antibodies with a limited activity against Beta and Omicron but induced an expanded antibody breadth overtime, up to 4.9 months post vaccination. In contrast, more than one third of RBD-binding IgG^+^ memory B cells with a resting phenotype initially bound the Beta and Omicron variants and steadily increased the B cell receptor (BCR) breadth overtime. As a result, a fraction of the resting memory B cell subset secreted Beta and Omicron-neutralizing antibody when stimulated in vitro. The neutralizing breadth of the resting memory B cell subset helps us understand the prominent recall of Omicron-neutralizing antibodies after an additional booster or breakthrough infection in fully vaccinated individuals.

## Teaser

Memory B cells elicited by two doses of mRNA vaccine preserve broadly neutralizing antibodies against Omicron variant.

## INTRODUCTION

SARS-CoV-2 mRNA vaccination elicits long-lived plasma cells and memory B (B_mem_) cells, which constitute two layers of the humoral protection against SARS-CoV-2 (*1*–*8*). The pre-existing antibodies provided by plasma cells prevent infection at the initial infection site and contribute to sterilizing immunity if they are present in sufficient amounts at the time of infection. However, the pre-existing antibodies gradually wane with time after vaccination with mRNA vaccines (*1*, *2*, *9*). SARS-CoV-2 virus or viral antigens are then recognized by the BCR of B_mem_ cells that differentiate into plasma cells as the second arm of humoral protection. Importantly, B_mem_ cells remain in the peripheral blood without noticeable decline for up to six months after two mRNA vaccine doses (*1*, *2*). Therefore, robust humoral protection by persistent B_mem_ cells along with T-cell-mediated cellular immunity is suggested to prevent the onset of symptomatic or severe disease in vaccinees (*10*), likely contributing to the long-lasting vaccine effectiveness for preventing severe disease (*10*).

The emergence of SARS-CoV-2 variants raises the issue of antibody evasion, as some of them carry many mutations in the receptor-binding domain (RBD) of the spike protein, the main target for potently neutralizing antibodies (*11*–*16*). Among the variants of concern that have emerged to date, the Beta and Omicron variants have a greater ability to escape from neutralizing antibodies than the Alpha and Delta variants (*17*–*22*). In particular, the Omicron variant profoundly escapes from the neutralizing activity of circulating antibodies in those who have received the second mRNA vaccine dose (> 23-122-fold reduction of neutralizing activities) (*18*–*22*). Furthermore, the Omicron variant is resistant to many therapeutic monoclonal antibodies that were clinically applied prior to the emergence of the variant (*18*, *20*, *23*–*25*). Epidemiological evidence further suggests a reduction in the effectiveness of SARS-CoV-2 vaccines against the Omicron variant (*26*).

Despite the antigenic alteration in the Omicron variant, the third dose of mRNA vaccine prominently induces neutralizing antibodies against the Omicron variant, inhibiting the loss of neutralizing activities (*18*–*22*). Furthermore, the breakthrough infection by non-Omicron variant robustly elicits Omicron-neutralizing antibodies in the vaccinees who have received mRNA vaccines (*27*–*29*). To gain mechanistic insights behind this phenomenon, it is important to dissect the B_mem_ cell response elicited by the second vaccine dose, focusing on the breadth against the variants and the neutralizing activity.

In this study, we examined the breadth of B_mem_ cells against the Beta and Omicron variants and the temporal shift in the composition of the B_mem_ cell subsets over time in those who have received two doses of mRNA vaccine. One of the persistent B_mem_ cell subsets with resting phenotype durably preserved the variant-reactive antibody repertoires at least up to 4.9 months after vaccination and about 30% of B_mem_-derived IgG broadly neutralized Beta and Omicron variants. Thus, these results suggest the potential contribution of this B_mem_ cell subset to the neutralizing breadth of recall antibodies after the third vaccine dose or breakthrough infection by a non-Omicron strain.

## RESULTS

### Study cohort

**Fig. 1.**
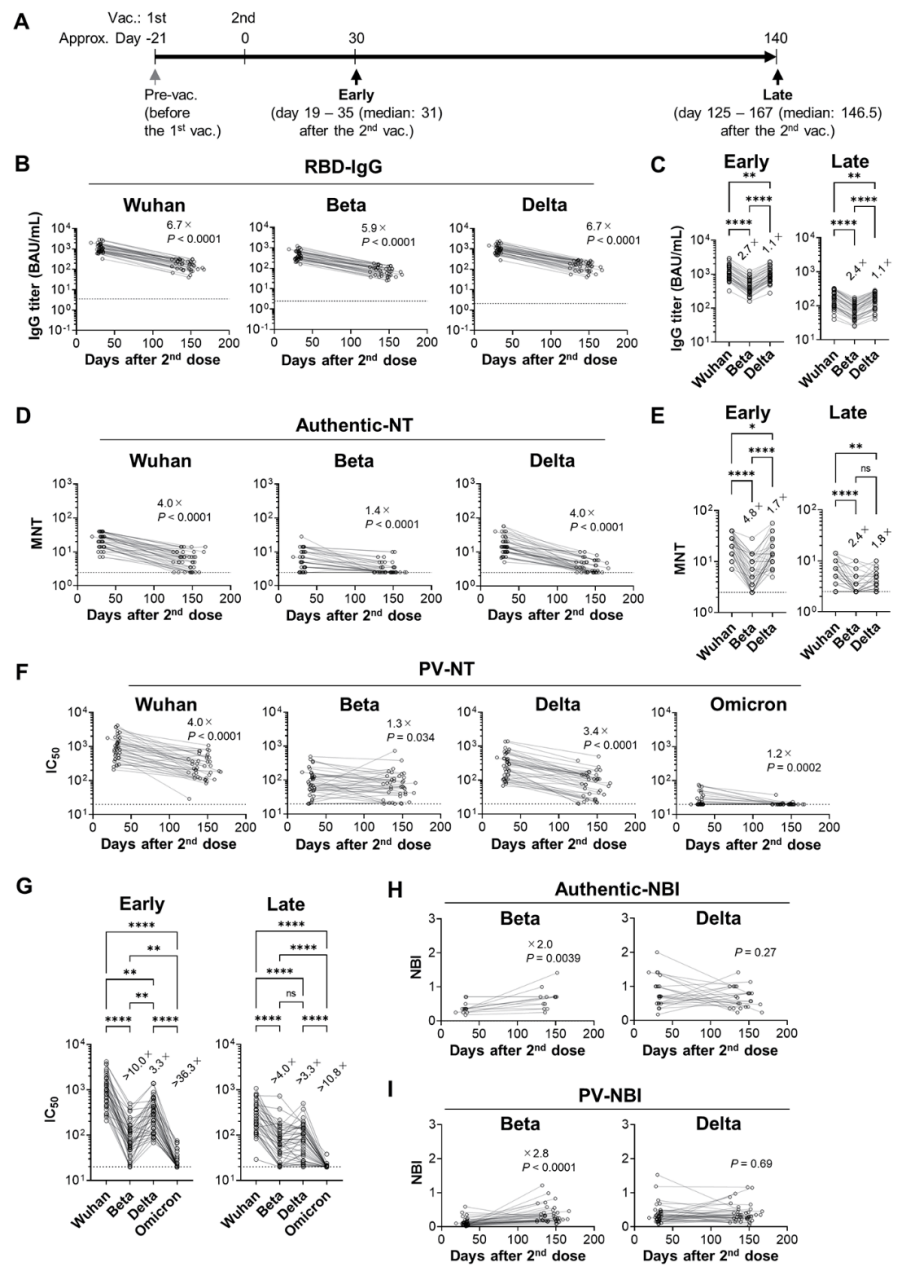
Longitudinal analysis of plasma neutralizing antibody. (**A**) Schematic of the sample collection is described. Plasma and PBMC were collected from female [*n* = 24, median age = 47 years (IQR: 36.25 - 51)] and male [*n* = 16, median age = 45.5 years (IQR: 35.25 – 57.5)] healthcare workers. (**B, C**) RBD-binding IgG titers to Wuhan, Beta, and Delta strains in plasma were quantified by ECLIA and expressed in WHO/NIBSC international antibody units (BAU)/mL. (**B**) Longitudinal analyses of the binding titer to each variant RBD are shown. The dotted lines indicate mean + 6 SD of the pre-vaccination plasma titer. (**C**) IgG titers to the variant RBDs are plotted. (**D, E**) NT titers were quantified using authentic viruses. The dotted lines indicate the limit of detection (2.5). (**D**) Longitudinal analyses of MNT are shown. (**E**) MNT titers to the variants are shown. (**F, G**) NT titers (IC_50_) were quantified using pseudoviruses. The dotted lines indicate the limit of detection (20). (**F**) Longitudinal PV-NT analyses are shown. (**G**) PV-NT titers (IC_50_) to the variants are shown. (**H, I**) Breadth of MNT (**H**) or PV-NT (**I**) to the variants were calculated by dividing the titers against the variant by the titers against the Wuhan and then plotted as neutralization breadth index (NBI). Samples were excluded when the NT titers below the detection limit at either the early or the late time points. Values on the plots indicate median of fold changes to the early time point (B, D, F, H, and I) or those to Wuhan strain (C, E, and G). Statistical analyses were performed with the Wilcoxon test (B, D, F, H, and I) or the Friedman test (C, E, and G: *P < 0.05, **P < 0.01, ***P < 0.001, ****P < 0.0001, n.s.: not significant). Data were pooled from three independent experiments (B – G; *n* = 40, H; *n* = 13 (Beta), 24 (Delta), I; *n* = 35 (Beta), 39 (Delta)).

We initially recruited 45 healthcare workers who received two doses of the Pfizer-BioNTech BNT162b2 mRNA vaccine for longitudinal blood donation. During the early (median = 31 days) and late (median = 146.5 days) time points after the second dose, the circulating antibodies and B_mem_ cells in the peripheral blood were analyzed by multiple parameters (Fig. 1A). Based on the prior history of COVID-19 diagnosis and anti-nucleocapsid antibodies in pre-vaccinated plasma, 40 volunteers were selected as naïve for further analysis (fig. S1A). The median age was 46.5 years, ranging from 25 to 73 years, and 16/40 volunteers (40%) were male (fig. S1B). No significant difference was observed in the median age between males and females.

### Temporal shift of variant-neutralizing activity of plasma antibodies

In COVID-19 convalescent individuals, neutralizing antibodies in plasma improve the breadth against the Beta variant over time, while the amounts of RBD-binding antibodies gradually decline (*30*, *31*). Here, we comparatively assessed the vaccine-elicited neutralizing antibodies using multiple serological parameters at the early and late time points (Fig. 1A). We focused on RBD, as it includes the major epitopes of neutralizing antibodies (*11*–*16*). The concentration of RBD-binding IgG and IgA antibodies in plasma were quantified by electrochemiluminescence immunoassay (ECLIA) and expressed as WHO/NIBSC international antibody units (BAU) per mL using a converting unit provided by a manufacturer. Consistent with previous reports, two doses of the mRNA vaccine robustly induced Wuhan RBD IgG titers, increasing at one month > 2000-folds above the level of pre-vaccination (Fig. 1B and fig. S2A). Although the Wuhan RBD IgA titers in plasma increased 44-folds after vaccination (fig. S2A), the abundance and correlation with neutralizing activities suggested a major contribution of IgG antibodies on the neutralizing activity of plasma antibodies (fig. S2B). The variant-reactivity of IgG antibodies against Beta and Delta was also determined by the validated ECLIA, showing slight but significant reduction for these variants (Fig. 1C).

The neutralizing antibody titers against the Beta and Delta variants were determined by neutralization (NT) assay using authentic viruses (MNT) and expressed as the endpoint titers that blocked the virus cytopathic effects (*30*). Although our method was applied in an international collaborative study (WHO/BS/2020.2403), the neutralizing titers were also assessed by VSV-pseudovirus (PV) assay for confirmation. For the Omicron variant, the neutralizing antibody titers were determined by PV-NT assay only, because the wider dynamic range of PV-NT assay allowed us to evaluate antigenic change in the Omicron variant more precisely. Neutralizing activities were more reduced by the variants than the binding activities of antibodies (Fig. 1, D and E). The neutralizing antibodies against the Beta variant declined more slowly than those against Wuhan and Delta, the phenomenon previously observed in the convalescent plasma (*30*). The slow decline of the Beta-neutralizing antibody was reproduced in the PV-NT assay (Fig. 1F). Neutralizing antibodies against the Omicron variant were more profoundly reduced than the other variants (Fig. 1G), precluding the calculation of the decay rates in Omicron-neutralizing antibody.

The neutralizing breadth of antibodies against the variants was assessed by neutralization breadth index (NBI) that represents the relative neutralizing activities to Wuhan strain versus those to the variants (*30*). The NBIs from Beta and Delta were calculated from the plasma samples above the detection limit, showing 2-fold (MNT) and 2.8-fold (PV-NT) increases against the Beta but not the Delta variant (Fig. 1, H and I). Thus, despite the quantitative decline of neutralizing antibody titers, we observed a steady increase in the neutralizing breadth against the Beta but not the Delta variant in the vaccinated plasma, suggesting that the declining antibody titers are not directly proportional to the loss of the neutralizing activities against the variant with antigenic change.

### Temporal shift of B_mem_ cell subsets

**Fig. 2.**
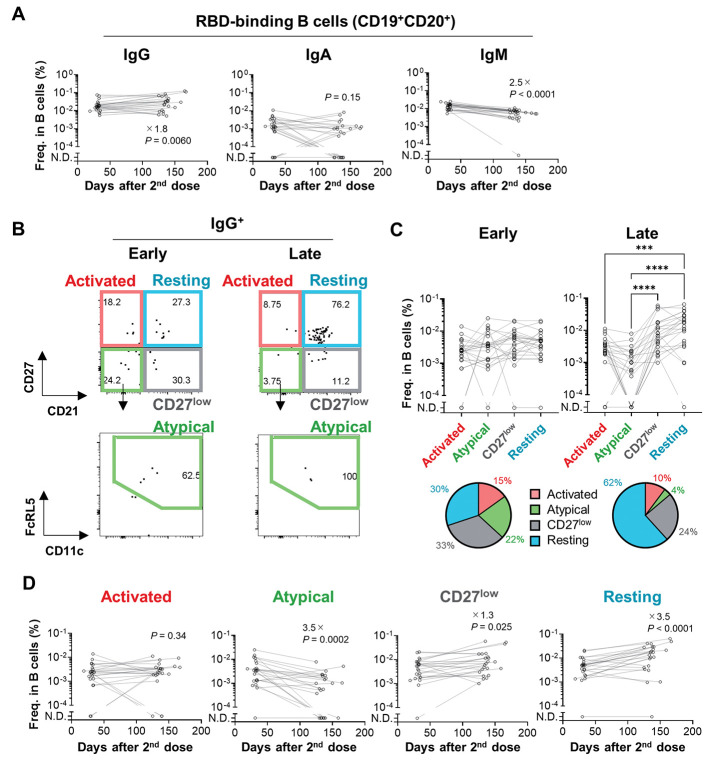
Longitudinal analysis of B_mem_ cell subset dynamics. (**A**) Longitudinal changes in the frequencies of Wuhan RBD-binding B cells (spike/RBD-binding CD19^+^CD20^+^ cells) expressing IgG, IgA, or IgM were enumerated using flow cytometry. (**B**) RBD-binding IgG^+^ B cells (spike/RBD-binding CD19^+^CD20^+^IgG^+^ cells) were subdivided into four B_mem_ subsets, including activated (CD21^-^CD27^high^), atypical (CD21^-^CD27^low^CD11c^+^FcRL5^+^), CD27^low^ (CD21^+^CD27l^low^), and resting (CD21^+^CD27^high^) B_mem_ cells. (**C**) The frequencies of the four B_mem_ subsets were determined as gated in (**B**). In the pie charts, frequency of B_mem_ subsets among RBD-binding IgG^+^ B cells were plotted. (**D**) Longitudinal changes in the frequencies of RBD-binding IgG^+^ B_mem_ subsets were analyzed. Values on the plots indicate fold changes of median. Statistical analyses were analyzed with the Wilcoxon test (A and D) or the Friedman test (C, ***P < 0.001, ****P < 0.0001). Data were pooled from two independent experiments (*n* = 23).

We then tracked the frequencies and phenotypes of RBD-binding B_mem_ cells in peripheral blood by flow cytometry. RBD-binding B cells were detected by simultaneous labeling with the Wuhan spike and RBD probes that were coupled to different fluorescent dyes. After gating on spike/RBD-binding CD19^+^CD20^+^IgM^-^IgG^+^ B cells, the cells were further subdivided into CD27^+^CD21^+^ (resting), CD27^low/-^CD21^+^ (CD27^low^), CD27^+^CD21^-^ (activated), and CD27^-^CD21^low^CD11c^+^FcRL5^+^ (atypical) (fig. S3) (*32*, *33*). Despite the quantitative decline of circulating IgG antibodies in the vaccinees, the numbers of RBD-binding IgG^+^ B cells increased 1.8-fold from the early to late time points (Fig. 2A), corresponding with previous reports (*1*, *2*). In contrast, IgA^+^ and IgM^+^ B cells were less persistent than IgG^+^ B cells (Fig. 2A). For IgM^+^ B cell, CD27^+^ B cells were further selected to avoid an inclusion of native B cells (fig. S4A). The CD27^+^ gating reduced the numbers 2.4-3.2-fold relative to those prior to the gating (Fig. 2A), but the IgM^+^ cells remained less persistent than IgG^+^ cells (fig. S4A). Hereafter, we focused on RBD-binding IgG^+^ B cells that were robustly induced and durably maintained after mRNA vaccination.

Four subsets in the RBD-binding IgG^+^ B_mem_ cells were separately enumerated based on surface markers (Fig. 2B). At the early time points, four subsets were comparably induced in the vaccinees (Fig. 2C). However, up to 4.9 months after vaccination, the number of resting B_mem_ subset increased by 3.5-fold, whereas the atypical B_mem_ subset decreased in numbers during the same time period (Fig. 2D), leading to the expanded composition of the resting B_mem_ subset among the IgG^+^ B_mem_ compartment in place of the reduction of the atypical B_mem_ subset. Although the numbers of RBD-binding IgG^+^ B_mem_ cells in human peripheral blood were relatively small, the resting B_mem_ subset expanded the ratio even after excluding the samples with less RBD-binding IgG^+^ B_mem_ cells (> 20 events per analysis) (fig. S4B). Furthermore, the spike-binding, RBD non-binding B_mem_ cells were subjected to the subset analysis (fig. S4C), reproducing the expansion of resting B_mem_ subset over time. Thus, the composition of spike-binding IgG^+^ B_mem_ cells, irrespective of RBD-binders or non-RBD-binders, changed over time, and the resting B_mem_ subset was the dominant subset at late time points.

### Broad-reactivity of resting B_mem_ subset against the variants

**Fig. 3.**
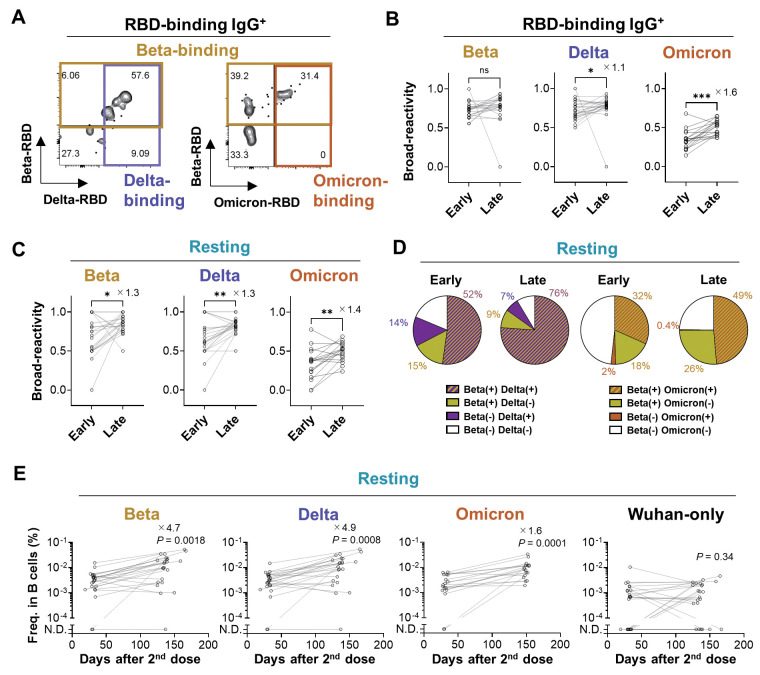
The variant-reactivity of the B_mem_ cell subset. (**A**) Wuhan RBD-binding IgG^+^ B_mem_ cells were subdivided into Beta- and Delta-binding (left) or Beta- and Omicron-binding (right) cells. (**B, C**) Frequency of the cells that bind the Beta, Delta, or Omicron RBDs among the Wuhan RBD-binding IgG^+^ B_mem_ cells (B) or the Wuhan RBD-binding IgG^+^ resting B_mem_ cells (C) were plotted. (**D**) The pie-charts show mean frequencies of each quadrant from Beta/Delta-binding (left) or Beta/Omicron-binding (right) among the Wuhan RBD-binding IgG^+^ resting B_mem_ cells at the early and the late time points. In Beta/Delta analysis, one donor without detectable resting B_mem_ cell was excluded from the analysis (*n* = 22, each time points). (**E**) Longitudinal changes in the numbers of IgG^+^ resting B_mem_ cells that bind Wuhan/Beta RBD (Beta), Wuhan/Delta RBD (Delta), Wuhan/Omicron RBD (Omicron), and Wuhan RBD only (Wuhan-only) were analyzed. Values on the plots indicate fold changes of median.Statistical analyses were performed with the Wilcoxon test (B, C, and E; *P < 0.05, **P < 0.01, ***P < 0.001, ns (not significant): *P* ≥ 0.05). Data were pooled from two independent experiments (Beta, Delta, and Wuhan only, *n* = 23; Omicron, *n* = 17).

To quantify the broad reactivity of B_mem_ cells against the variants, we prepared recombinant Omicron RBD protein. Since the Omicron variant harbors more than 5-fold higher numbers of RBD mutations than other variants, we assessed an antigenic structure of the Omicron RBD which is recognized by a reference antibody, S309 bearing the Omicron-neutralizing activity albeit at 3-fold reduced level (*18*). S309 bound the Omicron RBD with a slightly reduced affinity (fig. S5), comparably to the results of neutralizing activity (*18*). By using multiple RBD probes for labeling, the majority of RBD-binding IgG^+^ B_mem_ cells were broadly reactive against the Beta and Delta RBD variants (Fig. 3, A and B). Of note, Omicron reactivity was also detected in the one third of RBD-binding IgG^+^ B_mem_ cells at early time points. Given the limited breadth of plasma neutralizing antibodies (Fig. 1G) (*18*–*22*), the observed breadth to Omicron RBD in B_mem_ cells was noteworthy. The variant-reactivity was also detected in the resting B_mem_ subset with steady increase over time (Fig. 3C). As a result, the Omicron-reactivity was elevated from 38% to 52% in this subset. Intriguingly, the Omicron-binding B_mem_ cells were almost exclusively included in the Beta-binding population (Fig. 3D), possibly reflecting the overlapping of RBD mutations (K417N, E484K/A, and N501Y) among these variants. The increased breadth against the Beta, Delta, and Omicron RBDs was achieved by the prominent expansion of Beta-, Delta-, and Omicron-binding cells compared to Wuhan-specific cells (Fig. 3E).

### Omicron-neutralizing activities of B_mem_-derived monoclonal antibodies

**Fig. 4.**
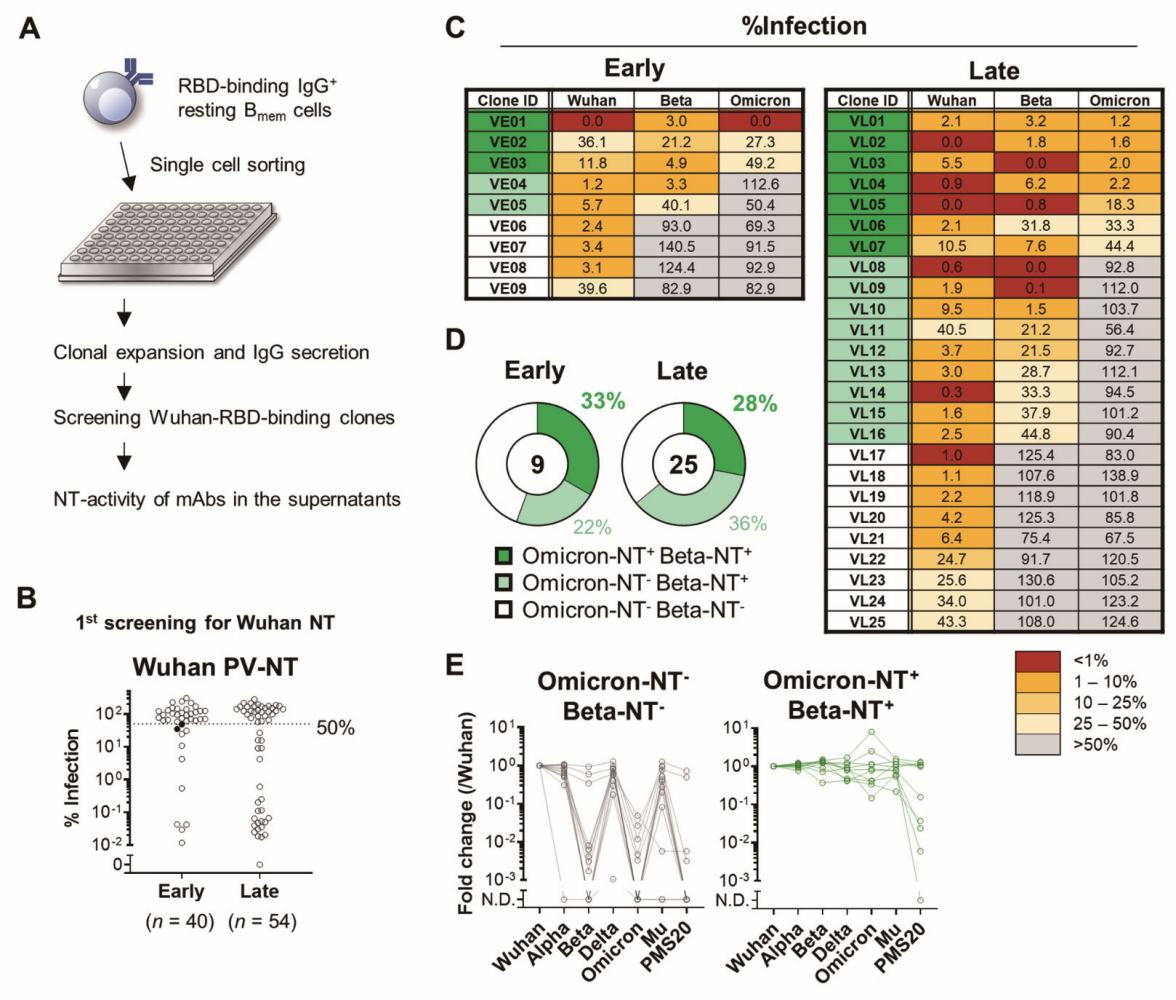
Broadly-neutralizing activity of B_mem_-derived monoclonal antibody. (**A**) Schematic diagram of the experimental workflow for assessing the neutralizing (NT) activity of monoclonal antibodies expressed by B_mem_ cells. In total, 229 monoclonal B cells were expanded and secreted >10 ng/mL IgG in the supernatants. Of those, 94 clones bound the Wuhan RBD in ELISA and further tested for NT activities to the Wuhan pseudovirus. (**B**) NT activities of individual monoclonal antibody at the first screening were plotted as percent infection calculated as the signals detected by individual antibody out of those without antibody. The antibodies with less than 50% of values were considered as positive for neutralizing activities. The two filled dots were excluded as the mean of three independent experiments exceeded 50% of cut-off value. (**C**) Mean % infection of monoclonal antibody clones from early (*n* = 9) and late (*n* = 25) time points of three independent experiments were indicated. (**D**) Pie charts represent the ratios of monoclonal antibody clones with Beta- and Omicron-neutralizing activity. (**E**) Binding breadth of monoclonal antibody clones listed in (C) was quantified with ECLIA. Signals to each RBD were normalized to those of reference antibody and fold changes to Wuhan RBD were calculated. Clones neutralizing Wuhan-only (Omicron-NT^-^ Beta-NT^-^, *n* = 13) and Wuhan/Beta/Omicron (Omicron-NT^+^ Beta-NT^+^, *n* = 10) were plotted separately.

The preservation of broadly reactive BCRs in the resting B_mem_ subset prompted us to examine the broadly neutralizing activity of IgG antibodies expressed by these cells. Flow cytometric analysis allowed us to quantify the percentages of RBD-binding B_mem_ cells with broad reactivity to the variant RBDs; however, the broadly neutralizing activities could not be assessed through this binding assay. To this end, RBD-binding IgG^+^ B_mem_ cells with a resting phenotype (*n* = 738) were sorted from a total of 23 donors for single-cell culture (Fig. 4A) (*34*). Owing to the paucity of RBD-binding IgG^+^ B_mem_ cells with resting phenotype, we used the pooled PBMC for sorting. Monoclonal IgG^+^ antibody clones secreted into the supernatants were then subjected to the binding assay by ELISA and to the PV-NT assay. We excluded IgG clones at less than 10 ng/mL IgG in the supernatants for detecting neutralizing activities above the limit of detection. From the selected IgG^+^ clones (*n* = 229), 94 clones bound monomeric RBD with high affinity above the limit of detection. An initial screening selected 36 clones as the Wuhan-neutralizing clones (Fig. 4B). Subsequently, 34 clones were confirmed to be the neutralizers (Fig. 4B). Thereafter, these IgG clones were subjected to PV-NT for assessing the broadly neutralizing activities against the Beta and Omicron variants. In analogy to the variant-reactivity of B_mem_ cells by flow cytometry, the substantial fraction of B_mem_-derived IgG retained the neutralizing activities to the Beta and Omicron variants (Fig. 4C). In total, 21 (59%) and 10 (27%) out of 34 antibodies neutralized Beta and Omicron variants, respectively (Fig. 4D). The distribution of broadly neutralizing antibodies at the early and late time points was roughly comparable (Fig. 4D), but the point needs to be carefully assessed by larger numbers of antibody panel. Again, the Omicron-neutralizing clones were confined to the Beta-neutralizing clones, as observed in the binding data (Fig. 3).

Finally, the binding breadth of the neutralizing antibody clones to a panel of variant RBDs were assessed by ECLIA. The PMS20 RBD mutant is designed to carry multiple mutations conferring resistance to multiple classes of neutralizing antibodies (*35*). All of Wuhan-specific neutralizing clones poorly bound to the Omicron RBD but most of them retained the binding to Alpha, Delta, and Mu variants (Fig. 4E). They failed to bind Beta RBD except three clones (VE08, VE09, and VL22) that bound Beta RBD without neutralizing activity (Fig. 4, C and E). Two clones (VE08 and VE09) further bound PMS20 RBD (Fig. 4E, left), showing the broad RBD binding in these clones except Omicron RBD. The partial mismatch between the binding and neutralizing activity was likely due to the better sensitivity in the binding assay than neutralization assay. Nonetheless, the binding of all Omicron^+^Beta^+^ clones to Beta and Omicron RBDs supported the broadly neutralizing activities. Moreover, half of the Omicron-neutralizing clones showed the strong binding to PMS20 as well (Fig. 4E, right). These results highlight the extreme breadth of Omicron-neutralizing clones that are durably preserved in the resting B_mem_ subset after mRNA vaccination.

## DISCUSSION

Long-lived plasma cells continuously secrete highly specific, high-affinity antibodies prior to infection (*5*, *6*), contributing to sterilizing immunity. However, the limited breadth of pre-existing antibodies permits profound escape of highly mutated Omicron variant, suggesting attenuation of vaccine effectiveness and increased risk of breakthrough infection. B_mem_ cells typically express lower affinity antibodies than those from plasma cells as a result of differential selection threshold (*36*, *37*). The less stringent selection is suggested to expand a breadth of B_mem_ cells as observed in multiple mouse models (*38*, *39*), but the clinical relevance of the B_mem_ breadth still remains unknown. Despite the usage of non-variant antigen for the mRNA vaccines currently used, they elicited Omicron-neutralizing B_mem_ cell subsets that occupied around 30% of cells among Wuhan-neutralizing B_mem_ cells. Omicron-neutralizing cells were not dominant population, but the frequencies might be sufficient to expand the neutralizing breadth of boosted antibodies after the third dose compared to the limited breadth that remains ineffective to the Omicron variant after the second dose. These results support the possible contribution of B_mem_ breadth to the broadly neutralizing antibody responses after an additional boosting by mRNA vaccine or non-Omicron breakthrough infection.

How broadly neutralizing B cells are selected and recruited into the B_mem_ compartment for increasing the B_mem_ breadth is not known. We observed that 38% of RBD-binding resting B_mem_ cells bound the Omicron RBD in the early time points and the percentages further increased to 52% in the late time points, showing that the B_mem_ breadth obtained by the early time points was further improved over time. RBD-binding B_mem_ cells continue to undergo affinity maturation from 1.3 to 5 months (*2*). Such continual B_mem_ cell evolution may be sustained by newly generated precursors from persistent GC responses in the mRNA vaccinees (*40*), as the B_mem_ cell evolution is accompanied by > 2-fold increase in the numbers of somatic hypermutations as well as the frequencies of B_mem_ cells (*2*). High numbers of mutations in the broadly reactive B_mem_ cells over the strain-specific B_mem_ cells suggest more marked evolution of the broadly reactive B_mem_ cells (*1*).

The observed B_mem_ breadth might be achieved by two mutually non-exclusive models. In the first model, broadly reactive B cells and strain-specific B cells are derived from different clonal origins. Two B cell origins exhibit distinct specificity by targeting either conserved epitopes for broad reactivity or variable epitopes for strain-specificity. Indeed, multiple RBD epitopes still remain unchanged in the Beta and Omicron variants and there are the neutralizing monoclonal antibodies that target such conserved epitopes for achieving broadly neutralizing activity (*18*). The second model requires the clonal origins shared between broadly reactive and strain-specific clones. As a result of affinity maturation driven by the non-variant RBD antigen, some of the progenies from strain-specific clones acquire affinity-matured BCRs and an ability to bind mutant RBD irrespective of amino acid changes. Indeed, longitudinal BCR repertoire analysis shows the presence of such clones that expand the breadth to non-Omicron variant over time (*1*, *2*). Since nearly all Omicron-neutralizing clones were included in those with Beta-neutralizing activity, we speculate that the neutralizing breadth to Omicron could be acquired as a result of affinity maturation in the similar clonal origins.

Besides the breadth of B_mem_ cells, B_mem_-extrinsic factors also contributed to the expansion of antibody breadth after the boosting. One of B_mem_-extrinsic factors is an epitope masking by the pre-existing antibodies. At the time of the third vaccine dose, RBD-binding IgG antibodies elicited by the second vaccine dose are circulating in the body, albeit at quantitatively declined level with time. If the pre-existing antibodies masks the immunodominant epitopes of the boosted antigens before stimulating B_mem_ cells, it elevates the relative antigenicigy of otherwise subdominant epitopes for broadly neutralizing antibodies, as shown in humoral responses after vaccination by *Plasmomdium falciparum* circumsporozoite protein (*41*). Therefore, the epitope masking could also contribute to expand the breadth of neutralizing antibody responses after an additional booster.

The study was limited to the analysis after the second vaccine dose and not extended to the third vaccine dose. Further longitudinal studies are warranted to address how broadly neutralizing B_mem_ cells respond to an additional booster and contribute to the recall of Omicron-neutralizing antibody responses. In addition, there are insufficient information to determine whether broadly neutralizing B_mem_ cells migrate to mucosal sites and participate in the humoral protection after the variant virus infection at the local sites.

However, recent clinical studies give insights in related to the broadly neutralizing B_mem_ cell responses following breakthrough infection in fully vaccinated individuals (*27*–*29*). In these studies, the antibody responses are tracked in breakthrough infection cases by non-Omicron variants. Despite the infection by non-Omicron variants, the breakthrough infection recalls broadly neutralizing antibody against multiple variants including Omicron, similarly to an additional booster. Therefore, our data argue for the possible contribution of vaccine-elicited B_mem_ cells to the broadly neutralizing antibody response following breakthrough infection or the third mRNA vaccine dose in patients who already have two doses of an mRNA vaccine.

## MATERIALS AND METHODS

### Study design

We profiled the phenotypes of vaccine-elicited B_mem_ cells and the binding and neutralizing activity of the B_mem_-derived antibodies against SARS-CoV-2 variants. We recruited 40 healthcare workers who had neither the prior history of COVID-19 diagnosis nor anti-nucleocapsid antibody before vaccination. Following the second dose of BNT162b2 mRNA vaccine, we performed longitudinal analysis on the neutralizing breadth of plasma antibody against the Beta, Delta, and Omicron variants based on virus neutralization assays. The surface phenotypes and binding breadth of BCRs on the B_mem_ cells were longitudinally tracked by flow cytometric analysis using the variant RBDs as probes. Finally, we prepared B_mem_-derived monoclonal antibody panel and determined the neutralizing breadth of the B_mem_-derived antibodies against the Beta and Omicron variants.

### Human samples

Longitudinal blood samples were collected at approximately 0, 51, and 161 days after the first vaccination with the BNT162b2 mRNA vaccine (Pfizer) from healthcare workers who received two doses of the vaccine at Tokyo Shinagawa Hospital, Tokyo Metropolitan Bokutoh Hospital, Japan Community Health Care Organization Tokyo Shinjuku Medical Center, and Showa General Hospital. Blood was collected in Vacutainer CPT tubes (BD Biosciences), followed by centrifugation at 1800 × g for 20 min. Peripheral blood mononuclear cells (PBMCs) were suspended in plasma and harvested into different tubes, followed by centrifugation at 300 × g for 15 min. After the plasma was transferred into another tube, the PBMC pellets were washed with PBS three times before cryopreservation in Cell Banker 1 (Zenoaq). The plasma samples were further centrifuged at 800 × g for 15 min and transferred into another tube to completely remove the PBMCs. The plasma samples were heat-inactivated at 56°C for 30 min before use. Nucleocapsid antibody was analyzed by cobas e411 plus (Roche) with Elecsys Anti-SARS-CoV-2 (Roche), and <1 was evaluated as seronegative according to the manufacturer’s instructions. The present study was approved by the Institutional Review Board of the National Institute of Infectious Diseases (#1321) and was performed according to the Declaration of Helsinki. All volunteers provided written informed consent prior to the enrollment.

### Recombinant RBD preparation

The original and variant RBDs were prepared as previously described (*30*). Briefly, the human codon-optimized DNA sequence encoding amino acids 331-529 of the SARS-CoV-2 spike (GenBank: MN994467) with an N-terminal signal peptide sequence (MIHSVFLLMFLLTPTESYVD) and C-terminal avi-tag and histidine-tag were cloned into the pCAGGS vector. The vectors encoding variant RBDs bearing N501Y mutation (Alpha strain), K417N/E484K/N501Y mutations (Beta strain), L452R/T478K mutations (Delta strain), G339D/S371L/S373P/S375F/K417N/N440K/G446S/S477N/T478K/E484A/Q493R/G496S/Q498R/N501Y/Y505H mutations (Omicron strain), R346K/E484K/N501Y mutations (Mu strain), R346S/K417N/N440K/V445E/L455R/A475V/E484K/N501Y mutations (PMS20) (*35*) were generated in the same frame. The vectors were transfected into Expi293F cells according to the manufacturer’s instructions, and recombinant proteins produced in the supernatant were purified using Ni-NTA agarose (QIAGEN). To biotinylate RBD proteins, the RBD expression vectors were co-transfected into Expi293F cells together with the secreted BirA-Flag plasmid (Addgene) in the presence of 100 μM biotin.

### Electrochemiluminescence immunoassay (ECLIA)

Plasma antibody titers for the variant RBDs were measured using v-plex and u-plex kits (Meso Scale Discovery) according to the manufacturer’s instructions. Briefly, plates precoated with RBDs (SARS-CoV-2 Panel 11, Meso Scale Discovery) were incubated with MSD Blocker A reagent at room temperature for 1 hour with rotation. The plates were washed with PBS supplemented with 0.05% Tween-20 (washing buffer) three times and incubated with samples diluted in MSD Diluent 100 (Meso Scale Discovery) at room temperature for 2 hours with rotation. The plates were washed with washing buffer three times, followed by incubation with sulfo-tag-conjugated anti-human IgG or IgA (Meso Scale Discovery) at room temperature for 1 hour with rotation, and then the plates were washed three times with the washing buffer. The plates were treated with MSD Gold read buffer B (Meso Scale Discovery) and electrochemiluminescence was immediately determined with MESOQuickPlex SQ 120 (Meso Scale Discovery). Plasma IgG titers were calculated using the reference standard (Meso Scale Discovery) and were further converted to WHO/NIBSC international unit (BAU/mL) using a converting unit (×0.0272) provided by a manufacturer. To obtain comparable titers of IgA to those of IgG, we normalized the IgA titers using a converting unit, which was calculated from the binding curves of CR3022 monoclonal antibodies prepared as human IgG1 and human IgA1 isotypes (*30*, *42*).

For the u-plex assay, we conjugated the biotinylated RBDs to linker proteins of u-plex development pack (Meso Scale Discovery), according to the manufacturer’s instruction. Briefly, the biotinylated proteins were incubated with the linker proteins at room temperature for 30 min followed by incubation with Stop solution at room temperature for 30 min. The linker-conjugated RBDs were mixed and added to u-plex plates. The plates were incubated at 4°C overnight and then washed with the washing buffer. Thereafter, the same procedure as the v-plex assay was performed for quantifying plasma IgG and monoclonal IgG antibodies.

### Neutralization assay

Neutralization assays were performed as previously described (*30*). For authentic viral neutralization, plasma samples were serially diluted (2-fold dilutions starting from 1:5) in high-glucose DMEM supplemented with 2% FBS and 100 U/mL penicillin/streptomycin, and were mixed with 100 TCID_50_ SARS-CoV-2 viruses, namely WK-521 (hCoV-19/Japan/TY-WK-521/2020, Wuhan strain), TY8-612 (hCoV-19/Japan/TY8-612/2021, Beta variant), and TY11-927 (hCoV-19/Japan/TY11-927-P1/2021, Delta variant), followed by incubation at 37°C for 1 hour. The virus-plasma mixtures were placed on VeroE6/TMPRSS2 cells (JCRB1819) seeded in 96-well plates and cultured at 37°C with 5% CO_2_ for 5 days. After the culture, the cells were fixed with 20% formalin (Fujifilm Wako Pure Chemicals) and were stained with crystal violet solution (Sigma-Aldrich). The mean cut-off dilution index with > 50% cytopathic effect from 2 to 4 multiplicate series was presented as the neutralizing titer.

For the pseudovirus-neutralization assay, VSV-pseudoviruses bearing SARS-CoV-2 spike protein were generated as previously described (*43*). Briefly, cDNAs encoding the spike proteins of SARS-CoV-2 viruses, including WK-521 (Wuhan strain), TY8-612 (Beta variant), TY11-927 (Delta variant), and TY38-873 (hCoV-19/Japan/TY38-873P0/2021, Omicron variant) were cloned into the pCAGGS expression vector with a 19 aa truncation at the C terminus of the spike. The plasmid vectors were transfected into 293T cells followed by infection with 0.5 MOI of G-complemented VSVΔG/Luc 24 hours after the transfection (*44*), and then the uninfected viruses were washed out. After 24 hours of culture, the supernatants with the VSV-pseudoviruses were harvested, centrifuged to remove cell debris, and stored at -80°C until conducting the neutralization assay. For the assay, the pseudoviruses were mixed with an equal volume of serially diluted plasma (5-fold dilutions starting from 1:10 dilution) or B cell culture supernatants diluted at 1:4 ratio, and were incubated for 1 hour at 37°C. The mixtures were inoculated on VeroE6/TMPRSS2 cells seeded in 96-well solid white flat-bottom plates (Corning) and incubated at 37°C for 24 hours in a humid atmosphere containing 5% CO_2_. Luciferase activities in the cells were measured using the Bright-Glo luciferase assay system (Promega) using a GloMax Navigator Microplate Luminometer (Promega). Half-maximal inhibitory concentrations (IC_50_) were calculated using Prism 9 (GraphPad).

### ELISA

ELISA was performed as previously described (*30*). Briefly, F96 Maxisorp Nunc-ELISA plates (Thermo Fisher Scientific) were incubated with 2 μg/mL of the recombinant RBD or anti-human IgG Fab at 4°C overnight. The plates were washed with PBS containing 0.05% Tween-20 and blocked with PBS supplemented with 1% BSA. After the blocking buffer was discarded, samples and standards were placed into the wells, and the plates were incubated for 2 hours at room temperature or overnight at 4°C. After washing the plates, HRP-conjugated goat anti-human IgG (Southern Biotech) was added to the wells, and the plates were incubated for 1 hour at room temperature After washing, the OPD substrate (Sigma) was added to the wells followed by stopping the reaction with 2N H_2_SO_4_, and the absorbance at 490 nm was determined with an Epoch2 microplate reader (Biotek).

### Bio-layer interferometry

Kinetic assays were performed by capturing recombinant biotinylated RBDs on Octet SA biosensors (Sartorius) for 1 min, followed by 2 min baseline step in 1 x Octet kinetics buffer (Sartorius), 3 min association step in S309 IgG1 diluted with 1 × kinetics buffer, and 5 min dissociation step in 1 × kinetics buffer. The binding signals were acquired on OCTET R8 (Sartorius).

### Flow cytometry

Avi-tag-biotinylated spike and RBD were incubated with subsequent fluorochrome-labeled streptavidin at 4:1.5 ratio overnight at 4°C as follows: spike with APC-streptavidin (Invitrogen), Wuhan RBD with PerCP-Cy5.5-streptavidin (BioLegend), Beta-RBD with BUV661-streptavidin (BD Biosciences), and Delta RBD or Omicron RBD with PE-streptavidin (Invitrogen). PBMCs were thawed at 37°C and immediately washed with DMEM supplemented with 2% FBS, followed by staining with the spike/RBD probes in DMEM supplemented with 2% FBS and 10 μM biotin for 30 min at room temperature. The cells were washed with the medium and stained with subsequent antibodies/reagents using the Brilliant Stain Buffer Plus (BD Biosciences) for 30 min at room temperature: FITC-conjugated anti-IgA (polyclonal rabbit F(ab’)2, Dako), BV421-conjugated anti-IgG (G18-145, BD Biosciences), BV510-conjugated anti-human CD2 (RPA-2.10, BioLegend), BV510-conjugated anti-human CD4 (RPA-T4, BioLegend), BV510-conjugated anti-human CD10 (HI10a, BioLegend), BV510-conjugated anti human CD14 (M5E2, BioLegend), LIVE/DEAD Fixable Yellow Dead Cell Stain kit (Thermo Fisher Scientific), BV605-conjugated anti CD27 (O323, BD Biosciences), BV650-conjugated anti-FcRL5 (509F6, BD Biosciences), BUV395-conjugated anti-CD19 (HIB19, BD Biosciences), BUV496-conjugated anti-CD20 (2H7, BD Biosciences), BUV563-conjugated anti-IgM (UCH-B1, BD Biosciences), BUV615-conjugated anti-CD11c (3.9, BD Biosciences), and BUV737-conjugated anti-CD21 (B-ly4, BD Biosciences). The cells were washed with DMEM supplemented with 2% FBS, followed by resuspension in the medium and flow cytometry with FACS Symphony S6 (BD Biosciences). Data were analyzed using FlowJo software (BD Biosciences).

### Single B cell culture

A single B cell culture, called Nojima-culture, was performed (*34*). Single RBD-reactive resting B_mem_ cells were sorted into 96-well plates containing RPMI1640 medium supplemented with 10% FBS, 55 μM 2-ME, 100 Units/mL penicillin, 100 μg/mL streptomycin, 10 mM HEPES, 1 mM sodium pyruvate, 1% MEM NEAA, 50 ng/mL recombinant human IL-2 (Peprotech), 10 ng/mL recombinant human IL-4 (Peprotech), 10 ng/mL recombinant human IL-21 (Peprotech), 10 ng/mL recombinant human BAFF (Peprotech), and pre-cultured MS40L-low feeder cells at one cell per well using FACS Symphony S6 (BD Biosciences). The plates were incubated at 37°C in a humid atmosphere with 5% CO_2_. The medium was half-replenished on days 4, 8, 12, 15, and 21, and the supernatants were harvested on day 24.

### Statistical analysis

The numerical data were statistically analyzed and visualized with GraphPad Prism 9 software (GraphPad). Paired data and matched multiple data were analyzed with Wilcoxon test and Friedman test, respectively. Unpaired data were analyzed with Mann-Whitney test. For multiple comparison after the analyses, Dunn’s multiple comparison test was performed. Differences with *P* values less than 0.05 were considered significant. *P < 0.05, **P < 0.01, ***P < 0.001, ****P < 0.0001, ns (not significant) *P* ≥ 0.05. All the “*n*” in the present study indicates the numbers of biological replicates.
