# Supplementary material for: SARS-CoV-2 Omicron-neutralizing memory B-cells are elicited by two doses of BNT162b2 mRNA vaccine
Source: Sci Immunol. 2022 Feb 3:eabn8590. doi: 10.1126/sciimmunol.abn8590 (PMC8939773; doi:10.1126/sciimmunol.abn8590)
Supplement: Supplementary file 1 — Figs. S1 to S5 [file sciimmunol.abn8590_sm.pdf]

Science Immunology
AAAS

Supplementary Materials for

SARS-CoV-2 Omicron-neutralizing memory B-cells are elicited by two doses of BNT162b2 mRNA vaccine

Ryutaro Kotaki *et al.*

Corresponding author: Yoshimasa Takahashi, ytakahas@niid.go.jp

DOI: 10.1126/sciimmunol.abn8590

The PDF file includes:

Figs. S1 to S5

Other Supplementary Material for this manuscript includes the following:

Table S1

SARS-CoV-2 Omicron-neutralizing memory B-cells are elicited by two doses of BNT162b2 mRNA vaccine

Authors: Ryutaro Kotaki[1†], Yu Adachi[1†], Saya Moriyama[1†], Taishi Onodera[1†], Shuetsu Fukushi[2], Takaki Nagakura[1], Keisuke Tonouchi[1], Kazutaka Terahara[1], Lin Sun[1], Tomohiro Takano[1], Ayae Nishiyama[1], Masaharu Shinkai[3], Kunihiro Oba[4], Fukumi Nakamura-Uchiyama[5], Hidefumi Shimizu[6], Tadaki Suzuki[7], Takayuki Matsumura[1], Masanori Isogawa[1], Yoshimasa Takahashi[1*]

Affiliations:

[1]Research Center for Drug and Vaccine Development, National Institute of Infectious Diseases; Tokyo 162-8640, Japan.

[2]Department of Virology I, National Institute of Infectious Diseases; Tokyo 162-8640, Japan.

[3]Tokyo Shinagawa Hospital; Tokyo, 140-8522, Japan.

[4]Department of Pediatrics, Showa General Hospital; Tokyo, Japan

[5]Department of Infectious Diseases, Tokyo Metropolitan Bokutoh Hospital; Tokyo, Japan

[6]Department of Respiratory Medicine, JCHO Tokyo Shinjuku Medical Center; Tokyo, Japan

[7]Department of Pathology, National Institute of Infectious Diseases; Tokyo 162-8640, Japan.

[*]Corresponding authors.

Yoshimasa Takahashi

Research Center for Drug and Vaccine Development, National Institute of Infectious Diseases

Email: ytakahas@niid.go.jp

†These authors contributed equally to this work.

SUPPLEMENTARY MATERIALS

Fig. S1. Participant demographics.

Fig. S2. RBD-binding antibody titers and correlation to neutralizing activities.

Fig. S3. Flow cytometry gating strategy for RBD-binding B_{mem} cells.

Fig. S4. Additional analyses on the vaccine-induced B_{mem} cell subsets.

Fig. S5. S309 antibody binding to Omicron RBD

Supplemental Table S1: Raw data file (excel)

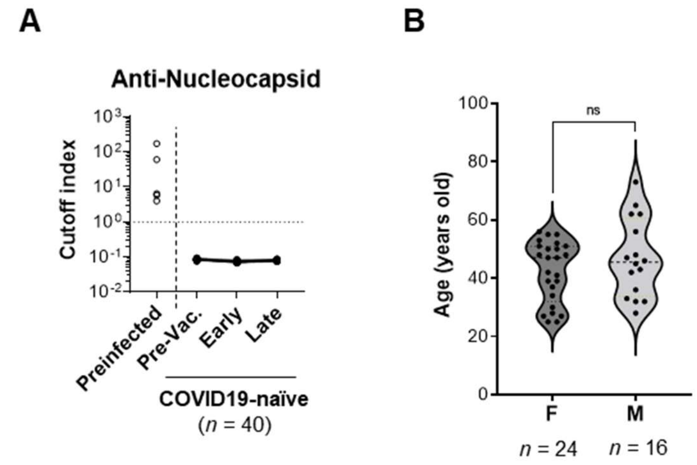

Fig. S1. Participant demographics.

(**A**) Anti-nucleocapside antibody titers of plasma collected prior to the first vaccination and at the early and late time points were measured. The dotted line on the y-axis indicates a detection limit (1.0) for the assay provided from the manufacturer. Samples with values above the detection limit at the pre-vac. time point were excluded from subsequent analyses. (**B**) Age distribution of the subjects was analyzed using the Mann-Whitney U-test (ns, not significant: $P \geq 0.05$).

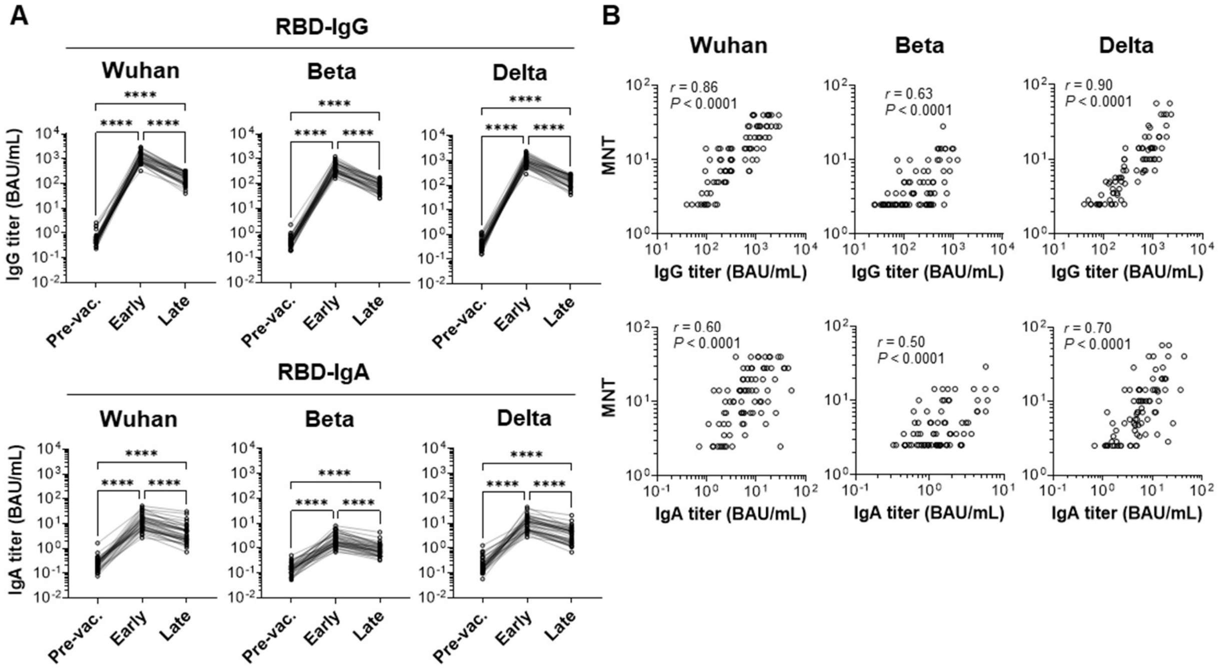

Fig. S2. RBD-binding antibody titers and correlation to neutralizing activities.

(**A**) Longitudinal RBD-binding IgG and IgA titers were measured using ECLIA. (**B**) Correlations between RBD-binding IgG or IgA and neutralizing titers were analyzed.

Statistical analyses were performed using the Friedman test (A) and Spearman's correlation analysis (B). ****$P < 0.0001$. Data were pooled from more than two independent experiments.

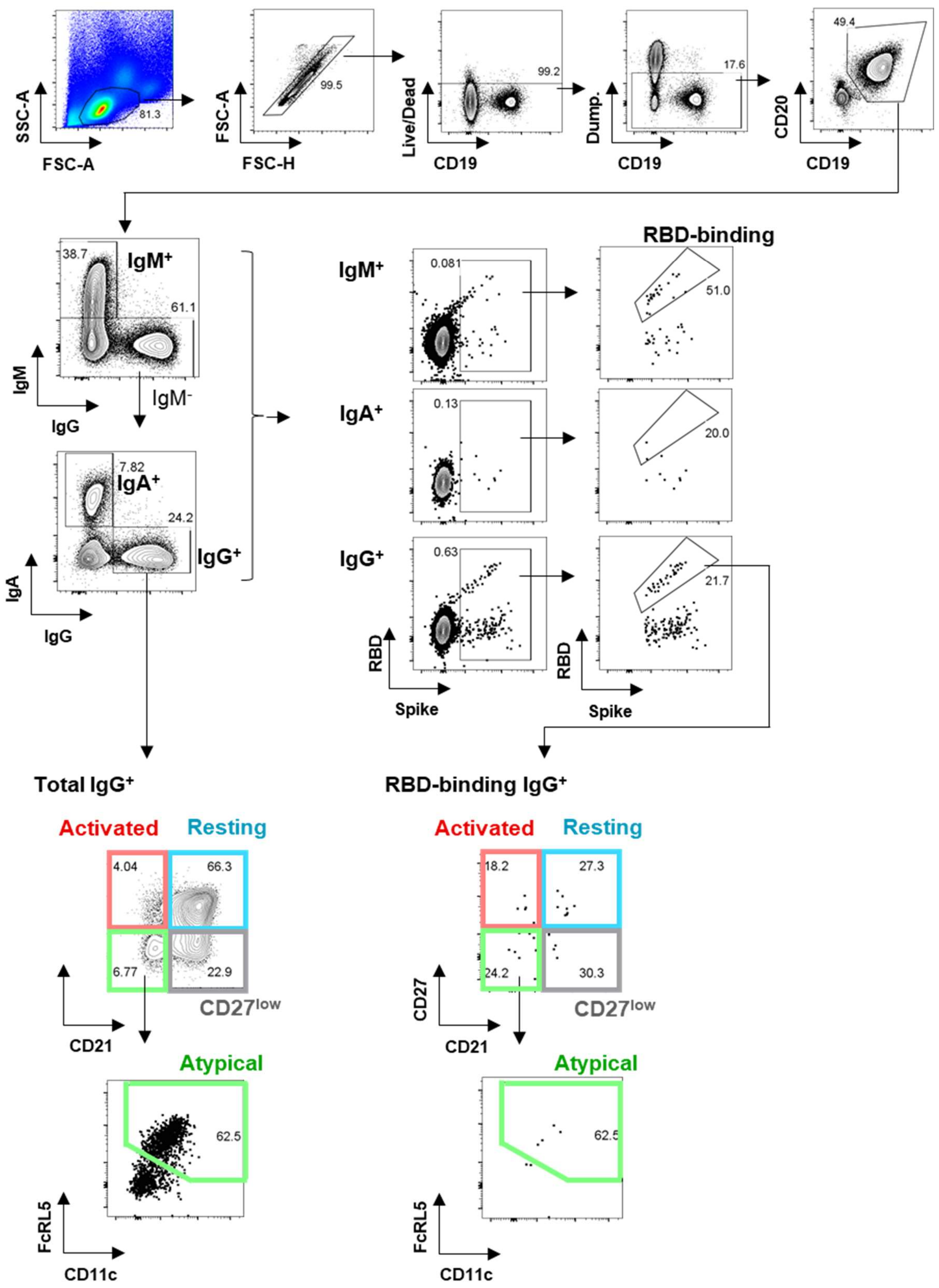

SSC-A
FSC-A
81.3
FSC-A
FSC-H
99.5
Live/Dead
CD19
99.2
Dump.
CD19
17.6
CD20
CD19
49.4
38.7
IgM+
61.1
IgM
IgG
IgM-
7.82
IgA+
24.2
IgG+
IgA
IgG
RBD-binding
IgM+
0.081
51.0
IgA+
0.13
20.0
IgG+
0.63
21.7
RBD
Spike
RBD
Spike
Total IgG+
Activated
Resting
4.04
66.3
6.77
22.9
CD27low
CD21
Atypical
62.5
FcRL5
CD11c
RBD-binding IgG+
Activated
Resting
18.2
27.3
24.2
30.3
CD27
CD27low
CD21
Atypical
62.5
FcRL5
CD11c

Fig. S3. Flow cytometry gating strategy for RBD-binding B_{mem} cells.

RBD-binding IgM/IgA/IgG B_{mem} cells were initially selected as $Dump^-CD19^+CD20^+$ cells and then divided into IgM^+, IgA^+, or IgG^+ cells. After gating on spike/RBD-binding cells, IgG^+ fractions were further delineated into B_{mem} subsets based on CD21, CD27, CD11c, and FcRL5 expression as indicated in the plot. activated ($CD21^-CD27^+$), resting ($CD21^+CD27^+$), $CD27^{low}$ ($CD21^+CD27^{low}$), and atypical ($CD21^-CD27^{low}CD11c^+FcRL5^+$) subsets. Representative plots from four independent experiments are shown.

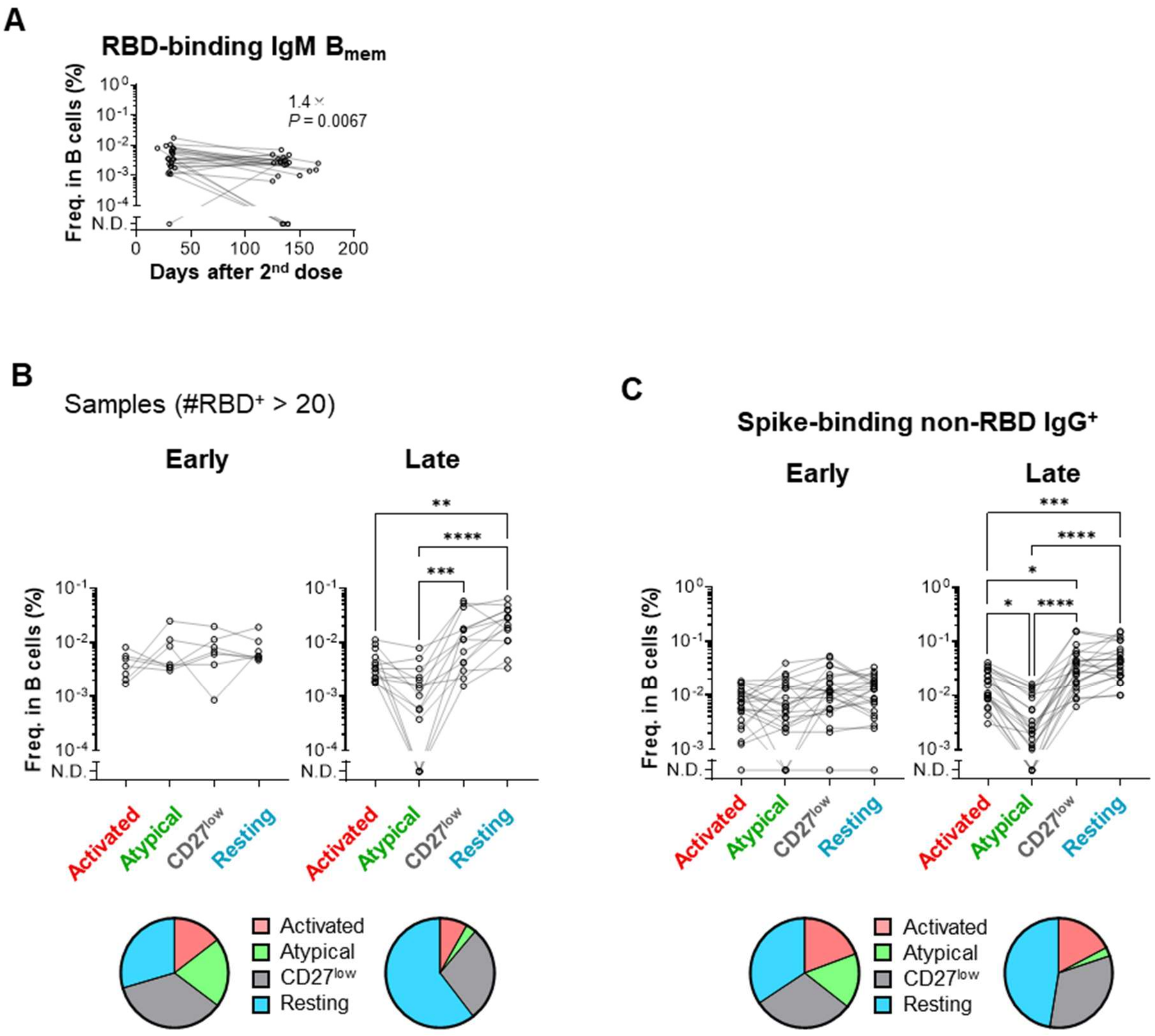

Fig. S4. Additional analyses on the vaccine-induced B$_{mem}$ cell subsets.

(**A**) Longitudinal frequencies of RBD-binding CD21^{+}CD27^{+} IgM B$_{mem}$ cells among CD19^{+}CD20^{+} B cells were analyzed (*n* = 23). Values on the plots indicate fold decrease of median from the early to the late time points. (**B**) B$_{mem}$ cell subsets were analyzed using samples with >20 RBD-binding IgG B$_{mem}$ cells. In the pie charts, median frequency of B$_{mem}$ subsets among RBD-binding IgG^{+} B cells were plotted (Early, *n* = 7; Late, *n* = 14). (**C**) B$_{mem}$ cell subsets were analyzed on Spike-binding, non-RBD-biding IgG^{+} B$_{mem}$ cells (*n* = 23). In the pie charts, median frequency of B$_{mem}$ subsets among RBD-binding IgG^{+} B cells were plotted. Statistical

analyses were performed with the Wilcoxon test (A), and the Friedman test (B, C) (*$P < 0.05$, **$P < 0.01$, ***$P < 0.001$, ****$P < 0.0001$). Data were pooled from two independent experiments.

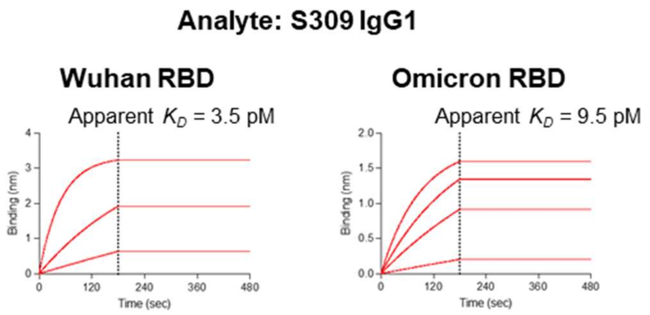

Fig. S5. S309 antibody binding to Omicron RBD

Binding of S309 IgG1 antibody to Wuhan-RBD (left) and Omicron-RBD (right) were quantitated with BLI. Biotinylated RBDs were captured on streptavidin-coated biosensors, and subsequently kinetics of S309 binding was analyzed. Signals were fitted to 1:1 binding model and apparent K_D values were calculated. Representative data from two independent experiments are shown.
